# Negative Valence Effect in Affective Forecasting: The Unique Impact of the Valence Among Dispositional and Contextual Factors for Certain Life Events

**DOI:** 10.5964/ejop.1945

**Published:** 2021-05-31

**Authors:** Virginie Christophe, Michel Hansenne

**Affiliations:** a PsyNCog Research Unit, Liège University, Liège, Belgium; University of Belgrade, Belgrade, Serbia

**Keywords:** affective forecasting, intensity bias, negative valence effect

## Abstract

Decades of research on affective forecasting have shown a persistent intensity bias—a strong tendency by which people overestimate their future hedonic response for positive events and underestimate it for negatives one. While previous research has provided answers on the isolated impact of various individual or contextual factors, this study is original in that it brings them together to determine which ones most influence the inaccuracy of affective forecasting. Participants were asked to predict their emotional satisfaction for a personal life event, the course (positive or negative) and date of which were already known. First, the results support previous research by showing that affective predictions are highly associated with people’s affective experience. Moreover, multiple regression showed that among the individual and contextual factors previously reported to be in relation with affective forecasting inaccuracy, only the valence of the event could explain inaccuracy of forecasting. According to a growing body of literature, these findings point out a tendency to underestimate the intensity of the affect predicted both for negative and positive, with a stronger underestimation for negative events: the negative valence effect.

## Affective Forecasting

Forecasting how we are going to feel in the future; that is what *affective forecasting* is.

Despite the adaptive role of our exceptional ability to mentally travel in time, several lines of evidence have showed that affective forecasting is subjected to many errors (e.g., Coteţ & David, 2016; Miloyan & Suddendorf, 2015; Wilson, Wheatley, Meyers, Gilbert, & Axsom, 2000). Affective forecasting bias mainly comes from people's tendency to overestimate their future hedonic response. For example, people could overestimate how happy they will be after positive events such as getting promoted and how sad they will feel after negative events, such as the loss of a match/election by a favorite sports team or political candidate. This tendency to overestimate the intensity of future emotion was first called *intensity bias* (Buehler & McFarland, 2001). Later, the overestimation of both intensity and duration errors were termed *impact bias* (Wilson, Meyers, & Gilbert, 2003): in short, people think that they will be impacted by stronger emotions and for a longer period than initially imagined. These biases are robust and have been observed in many diverse life events (e.g., Valentine's Day, political election, soccer, romantic breakup, or Christmas Day).

Although the intensity bias is considered as a robust phenomenon, recent studies have pointed out some methodological problems behind the studies shedding light upon it, and others have demonstrated that both dispositional and contextual factors could modulate it (Hoerger, Chapman, & Duberstein, 2016; Levine, Lench, Kaplan, & Safer, 2012, 2013;Verner-Filion, Lafrenière, & Vallerand, 2012).

## Different Approaches in the Study of Affective Forecasting

Recently, some authors have raised concerns about the usual means of measuring and interpreting affective forecasting inaccuracies, challenging the significance and validity of the intensity bias (Lench et al., 2019; Levine et al., 2012, 2013; Mathieu & Gosling, 2012).

A main concern that directly challenges the intensity bias is relative to a procedural artifact. Thus, the inaccuracy or the accuracy of our affective forecasting could be considered as being two facets of the same reality according to the choice of the data analyses (Dunn & Laham, 2006; Epley & Dunning, 2006; Gagné & Lydon, 2004). Convinced that the accuracy of affective forecasting can indeed be approached in several ways, Mathieu and Gosling (2012) conducted a meta-analysis. They explained that when researchers study the accuracy of forecasting via the subtraction of the affective forecasted score with the affective experienced score, the result obtained can be qualified as *absolute*. It reveals by “how much” we over- or underestimated our future affect, if at all. However, when the accuracy of the projections is studied through a correlation analysis between the predicted and the experienced score, the result obtained can be qualified as *relative*. It reveals the global direction of the forecasting error and reveals whether the trend of all the participants is leaning towards an overestimation or rather towards an underestimation of the forecasted affects. Their results showed that when accuracy was computed in *the absolute sense*, people tended to misestimate the absolute intensity of the hedonic response that they will experience, meaning that forecasters inaccurately predict exactly how happy positive events will make them and how unhappy negative events will make them. But, when accuracy was computed in *the relative sense*, the direction of people’s forecasts was reasonably accurate, meaning that forecasters accurately predict which positive events will make them happy and which negative events will make them unhappy. Thus, when researchers simultaneously consider the accuracy of forecasting in the absolute and the relative sense, it appears that, people can be seen as both good or bad predictors.

## The Previously Demonstrated Impact of Some Dispositional and Contextual Factors on Affective Forecasting

In order to better understand the conditions of occurrence of the intensity bias, a group of studies explored the impact of several factors on people’s affective forecasting (e.g., personality traits, event valence, importance or emotional intelligence). The purpose of these studies was to point out whether individual traits or contextual factors may lead individuals to overestimate or underestimate their future emotions, in other words, *who* are the people and *which* are the contexts favorable to the errors of affective forecasting?

### The Personality Traits and the Accuracy of Prediction

First, several studies focused on individual traits reported evidence supporting the link between personality traits and affective forecasting (e.g., Hoerger & Quirk, 2010; Zelenski, Santoro, & Whelan, 2012). Recently, Hoerger et al. (2016) showed that neuroticism and extraversion personality traits can be associated to both predicted and experienced affect for upcoming various life events (e.g., Valentine’s Day, football games, and birthday), meaning that personality modulates affective forecasting. More specifically, individuals who were lower in extraversion and higher on neuroticism anticipated, correctly, that they would experience relatively more unpleasant emotional reactions, and those who were higher on extraversion and lower on neuroticism predicted, rightly, that they would experience relatively more pleasant emotional reactions.

### The Level of Optimism and the Intensity Bias

Previously, research indicated that people believe unrealistic optimism to be preferable to realism (Armor, Massey, & Sackett, 2008) and exhibit optimistic biases about their personal abilities and their future (for a review, see Dunning & Story, 1991). Despite these suggestive findings indicating that people might assume that they will feel better than they end up doing in reality, only one published study directly examined the optimistic trait on affective forecasting. Indeed, Quoidbach and Dunn (2010) showed that the dispositional trait of optimism influenced the intensity bias for a positive event (i.e., 2008 victory of Obama). Ironically, the results showed that more optimistic individuals were less likely to see their emotional future positively.

### The Emotional Intelligence Competencies and Accuracy in Affective Forecasting

According to Hoerger, Quirk, Chapman, and Duberstein (2012), emotional intelligence could serve as a theoretical framework for organizing research on the influence of individual differences in affective projections. While previously, Dunn, Brackett, Ashton-James, Schneiderman, and Salovey (2007) showed that management of emotion—a subcomponent of a performance measure of emotional intelligence—emerged as the strongest predictor of forecasting ability, Hoerger et al. (2012) provided further proof that high emotional intelligence is associated with more accuracy for both performance and self-reported tests. By controlling participants' cognitive skills in addition to emotional intelligence, they asked students to predict the intensity of the emotions they would feel when viewing images that were first described to them. The results revealed that even with cognitive skill measures included in the model, emotional intelligence accounted for some of the variance of the forecasting accuracy score. Also, the authors demonstrated that emotional intelligence was associated not only with the accuracy of emotional projections, but also to their improvement across participants’ experience. Thus, the literature provides a series of studies suggesting that high emotional competencies improve affective forecasting accuracy (Dunn et al., 2007; Gasper & Clore, 2000; Gohm, 2003; Hoerger et al., 2012), just as personality or the level of optimism can modulate the predicted and experienced affect.

### The Direct or Indirect Consideration of the Contextual Valence in Literature

Many studies have examined the role of the valence of the event on affective prediction through its direct or indirect “positive” or “negative” categorization. For example, Christophe and Hansenne (2016) focused on the influence of *dispositional happiness* on affective forecasting accuracy, through different levels of success at an event (i.e., good, moderate or bad results in an exam). They showed that happy participants predicted less negative emotional feelings than unhappy people for moderate results, but no differences occurred between the two groups for good and bad results (i.e., an arbitrary positive and negative valence). Also, Hoerger and Quirk (2010) showed that neuroticism was associated with the forecasted and experienced affect in participants who did not have a Valentine's Day date (i.e., negative valence), but was only associated with experienced affect for those having had one (i.e., positive valence).

Through its direct or indirect consideration, the valence was one of the most common factors found in the literature about affective forecasting. To our knowledge, it was the only contextual factor to have been studied along with many other dispositional factors in the affective forecasting accuracy literature. Although research has shown that both positive and negative valences can impact the prediction of affect and accuracy, most showed a stronger effect for negative events (e.g., Andrade & Van Boven, 2010; Coteţ & David, 2016; Feys & Anseel, 2015; Finkenauer, Gallucci, Van Dijk, & Pollmann, 2007; Loewenstein, 2007; Mathieu & Gosling, 2012; Wilson, Meyers, & Gilbert, 2001).

### Studies About the Impact of the Subjective Importance of an Event

While dispositional traits and the valence were the first factors to be investigated to try to identify what might increase or decrease inaccuracy in affective forecasting, other contextual factors were subsequently explored. Few studies have investigated the implication of subjective importance given by participants to the event for which affective forecasting was asked (Eastwick, Finkel, Krishnamurti, & Loewenstein, 2008; Hoerger, Quirk, Lucas, & Carr, 2010; Verner-Filion et al., 2012) and little research has been published. The results showed that when the subjects have moderate expectations regarding the outcome of the event (e.g., moderate expectations in love or for a football team), the intensity bias is lowered compared to important expectations where the intensity bias is stronger.

### The Controversial Role of the Familiarity of an Event

Could the fact that we have already experienced an event improve the accuracy of forecasting? Previous studies have highlighted the relationships between memory and predictions (Levine, Lench, Karnaze, & Carlson, 2018), showing that we anticipate future affects based on memories of similar past events. Remembering is not as exact as one might think because of several recollection errors (Gilbert & Wilson, 2007; Morewedge, Gilbert, & Wilson, 2005; Yonelinas & Ritchey, 2015). However, in a meta-analysis, Coteț and David (2016) did not find any effect of familiarity (i.e., familiar vs. unfamiliar vs. unknown) as a moderator on the accuracy of affective predictions. In order to study this factor more precisely, a continuous variable like the frequency would be more appropriate.

## The Present Investigation

While many individual and contextual explanatory factors have been investigated mainly separately in affective forecasting research, few studies—if any—have yet studied them together. With regards to theoretical contribution, to the best of our knowledge, the present study provided the first piece of evidence examining which of these factors are the strongest predictors of affective forecasting inaccuracy. Considering the two distinct ways of approaching the accuracy of affective forecasting, we propose to explore both the absolute and relative forecasting accuracy through different analyses.

The first step will be to assess the relative accuracy between the predicted and experienced scores with a correlation analysis. Following Mathieu and Gosling (2012), we expect that participants will accurately predict their affects in the relative sense.

Then, to answer our main question, we will include in the same analysis: extraversion, neuroticism, optimism and emotional intelligence as dispositional factors, and the subjective importance of the event, the valence and frequency as contextual factors to explore which of them predict inaccuracy in affective forecasting for a likely life event. According to the predictors detected by the multiple regression model, we will know which contextual or dispositional factors most impact the absolute accuracy of affective forecasting for a personal life event.

Outside of valence, there is little data on the combined impact of contextual and individual factors on affective forecasting. Therefore, no assumptions can be made about the prevalence of some predictors over others. However, given the robustness of the valence found in multi-factor research, we expect that its effect will persist alongside other factors in our analyses.

## Method

### Participants

A total of 335 participants was recruited in the main hall of the university hospital. They were asked to participate in a study on positive psychology. The main investigator of the study informed them that they would be asked to answer a series of online questionnaires on a tablet for 30 minutes. After providing informed consent, demographic measures of age, gender and highest level of diploma (ranging from 1 to 7 for primary school degree, lower secondary, high school, bachelor, master, PhD and honorary degree) were asked. Additionally, the current affect was measured by the Positive and Negative Affect Schedule (PANAS; Watson, Clark, & Tellegen, 1988; French adaptation by Gaudreau, Sanchez, & Blondin, 2006) to control for the possible influence of their current affective state on our measure of interest (i.e., the inaccuracy score of affective forecasting). Participants completed the scales assessing both positive (10 items, α = .79) and negative affect (10 items, α = .85) on a 5-point Likert Scale ranging from *very slightly* to *very much*.

### Measures

*Extraversion* and *Neuroticism* were assessed by the Extraversion (8 items, α = .83) and Neuroticism (8 items, α = .84) subscales from the French version of Big Five Inventory (BFI-Fr; John & Srivastava, 1999; French adaptation by Plaisant, Courtois, Réveillère, Mendelsohn, & John, 2010). Participants indicated the degree to which the brief descriptive statements apply to them on a 5-point scale ranging from *strongly disagree* to *strongly agree*.

*Emotional intelligence* was assessed by the Trait Emotional Intelligence Questionnaire-Short Form (TEIQue-SF; Petrides & Furnham, 2006). The TEIQue-SF consisted of 30 items (α = .92) arranged on a 7- point response scale (from *strongly agree* to *strongly disagree*).

*Optimism* was measured by the French version of the Life Orientation Test–Revised (LOT–R; Scheier, Carver, & Bridges, 1994; French adaptation by Trottier, Mageau, Trudel, & Halliwell, 2008), which consists of 10 5-point items (α = .76).

*Dispositional happiness* was assessed by the French version of the Subjective Happiness Scale (SHS-F; Lyubomirsky & Lepper, 1999; French adaptation by Kotsou & Leys, 2017), which includes four 7-point items (α = .83).

### Procedure

#### Events and Contextual Measures

In the present study, participants were given the freedom to predict their feelings about a personal and likely event (for additional design information, see SM1 in Supplementary Materials) that they shared with the main investigator. Although the target event was not the same for all participants, some standardized instructions were given to them in order to frame their choice.

##### Valence

In previous research we have seen that the valence of the event can be arbitrarily attributed by researchers (e.g., Christmas Day as a positive event and no Valentine's Day date as a negative event). However, two people can attribute a different valence to the very same event depending on their ethnicity, age, needs, past experiences, life goals and personal characteristics. In the present study, participants were randomly instructed to give either a positive or a negative personal future life event. Through this procedure, we ensured that the valence data were in accordance with the subjective valence of the participant.

##### Occurrence

Considering the role of the likelihood of the outcome of an event on the accuracy of affective forecasting (Andrade & Van Boven, 2010; Ayton, Pott, & Elwakili, 2007; Buechel, Zhang, & Morewedge, 2017; Buehler & McFarland, 2001; Buechel, Zhang, Morewedge, & Vosgerau, 2014), we decided to control its variability. The event chosen could not be an event with a random outcome (e.g., football match, response to a job interview, medical examination results). The investigator made sure that the participants gave an event outcome that had an extremely high likelihood. Thus, a variety of personal life events was given by participants such as signing for the purchase of a new home, going to a dinner at their mother-in-law's house, celebrating their grandson's first birthday or having to announce poor diagnostic medical results to family members.

##### Event timing

Finally, the event should fulfill two timing conditions: it should be scheduled on a specific date already known at the time of answering the questionnaire and, occurring 4 weeks after predicting the affect. A tolerance of 2 days around the 4-week delay was granted in order to give the participants the opportunity to find an event that was already scheduled. Like the occurrence, event timing was therefore a fixed factor common to the entire sample. Only the valence, as a dichotomous variable, presented categories and was therefore included in our analyses as a contextual factor.

Once the personal event had been selected, the subsequent contextual measure of *subjective importance* and *past frequency* of the chosen event were asked for. The subjective importance was assessed on a 10-point Likert scale and the following frequency question was presented: “How many times have you experienced this event in the past?” Because of its large dispersion, frequency was converted to logarithmic scores.

#### Affective Forecasting

Time 1- Participants were asked to perform a forecast of the level of their emotional satisfaction about the event (for detail about the choice of the affective target, see SM2 in Supplementary Materials). The following instruction was presented: “Shortly after the end of this event, how do you think you will feel about it on a scale of 1 (*extremely bad*) to 10 (*extremely good*)?”

Time 2- A month later, the event for which affect had been predicted occurred. The experienced emotion was evaluated by phone within an 8-hour delay following the event. After having been asked if the event had occurred, participants were requested to rate their current affective state regarding the event on the same Likert’s scale. Following previous warnings found in the literature on affective forecasting about the method (see, Levine et al., 2012), a specific question about the experienced affect was presented (for additional information about metric issue in affective forecasting literature, see SM3 in Supplementary Materials): “Today was the day of the event you had previously forecasted. On a scale of 1 (*extremely bad*) to 10 (*extremely good*), how do you currently feel about it?”

Past research on affective forecasting has been based on a conceptualization of accuracy as the mathematical difference between the forecasted and the actual affect (Mathieu & Gosling, 2012). This method was presently used, and allowed us to obtain a measure of affective forecasting accuracy for each participant in order to study its potential modulation according to independent factors (e.g., Sevdalis & Harvey, 2007; Verner-Filion et al., 2012). Thus, the experienced affect score was subtracted from the forecasted affect score. A positive accuracy score indicated overestimation of the level of emotional satisfaction, a negative accuracy score showed its underestimation and a score of zero reflected exact prediction of the level of emotional satisfaction.

## Results

### Preliminary Analysis

Ninety-one percent of the 335 individuals accepted to participate. We excluded one participant who did not answer to the recall concerning experienced emotion, six for whom the event had not occurred, and four for whom the event that the participants actually experienced was not the one initially chosen. Analyses were conducted on a final sample composed of 295 individuals.

Prior to the main analysis, a preliminary analysis was conducted to determine whether the data was suitable for the analysis to be performed. Kurtosis statistics were used to examine normality assumption. According to Curran, West, and Finch (1996), the data are normally distributed if kurtosis values fall within the range of +1 and −1. Table 1 shows means, standard deviations, and kurtosis values with corresponding standard errors for each of the variables used in the study. As seen in Table 1, all variables were distributed within the range of +1 and −1, proving normal univariate distribution. However, the two dichotomous variables of gender and valence showed a platykurtic distribution due to their intrinsic non-metric properties.

**Table 1:** Descriptive Statistics of Study Variables (*N* = 295)

Variable	Range	*M*	*SD*	Kurtosis
1. Gender	0 - 1	0.45	0.498	−1.97
2. Age	16 - 86	40.83	15.31	−0.901
3. Education level	1 - 7	3.67	1.6	−0.805
4. Positive affect	12 - 40	27.76	4.84	−0.188
5. Negative affect	11 - 41	27.42	4.91	0.163
6. Extraversion	1.37 - 5	3.22	0.731	−0.445
7. Neuroticism	1 - 4.75	2.83	0.823	−0.698
8. Emotional intelligence	92 - 197	144	19.08	−0.132
9. Optimism	1 - 24	14.4	4.22	−0.059
10. Dispositional happiness	8 - 28	19.9	3.72	0.468
11. Valence	−1 - 1	0.105	0.996	−1.96
12. Importance	1 - 10	5.98	2.82	−0.997
13. Frequency	0 - 3.6	0.937	0.823	−0.143
14. Predicted score	1 - 10	6.99	1.95	0.208
15. Experienced score	1 - 10	7.49	1.97	0.214
16. Inaccuracy score	−5 - 4	−0.51	0.498	0.698

### Correlation Analyses

Pearson correlation coefficients were estimated among all the study variables assessed in the present study. Table 2 presents the correlation coefficients between the study variables. Results showed that predicted levels of emotional satisfaction were significantly correlated with the experienced level of emotional satisfaction, meaning a strong accuracy of forecasting in the relative sense. Also, Table 2 showed that the predicted and experienced scores were correlated with the inaccuracy score, with a positive association for the predicted emotional satisfaction and a negative association for its experience. No relationship between the inaccuracy score and any other factor was found. All individual variables of extraversion, neuroticism, emotional intelligence, optimism and dispositional happiness shared significant inter-correlations.

**Table 2:** Correlations Among the Study Variables (*N* = 295)

Variable	1	2	3	4	5	6	7	8	9	10	11	12	13	14
1. Age	−													
2. Education level	−.134*	−												
3. Positive affect	−.044	−.009	−											
4. Negative affect	−.034	−.003	.804***	−										
5. Extraversion	−.004	.019	.146*	.054	−									
6. Neuroticism	−.152**	.021	−.175**	.216***	−.201***	−								
7. Emotional intelligence	.025	.159**	.007	−.080	.514***	−.534***	−							
8. Optimism	.098	.139*	.002	−.066	.299***	−.389***	.511***	−						
9. Dispositional happiness	.056	−.006	.033	−.087	.318***	−.489***	.520***	.535***	−					
10. Importance	.192**	−.178**	.238***	.213***	.107	−.077	−.009	.069	.050	−				
11. Frequency	.170**	−.004	−.080	−.102	−.054	−.002	−.011	−.033	−.068	−.103	−			
12. Predicted score	.213***	−.122*	.035	.030	.049	−.152**	.103	.140*	.190***	.268***	−.027	−		
13. Experienced score	.164**	−.158**	.034	.069	.101	−.146	.138*	.147*	.206***	.242***	−.090	.700***	−	
14. Inaccuracy score	.062	.049	.001	−.051	−.068	−.006	−.047	−.012	−.023	.031	.082	.375***	−.399***	−

*p < .05. **p < .01. ***p < .001.

### Regression Analyses

In order to find which dispositional and contextual factors primarily impact the inaccuracy of affective forecasting, a multiple regression model was generated. This procedure allows for multicollinearity between some of the predictors to be handled (see Table 2) and to determine which independent variables most predict the absolute score of forecasting inaccuracy. The multicollinearity assumption was assessed during data analysis, through the tolerance statistic test and the Variance Inflation Factor (VIF) to demonstrate the absence of multicollinearity among independent variables. Tolerance values typically range from 0 to 1 (Mertler & Vannatta, 2005). Tolerance values less than .1 and VIF values greater than 10 indicate multicollinearity. Table 3 shows no evidence of multicollinearity in the study.

**Table 3:** Multiple Regression Model of Predictors on Inaccuracy Score of Forecasted Emotional Satisfaction

Dependent variable	Unstandardized β	*SE*	Standardized β	*p*	Collinearity statistics	
					Tolerance	VIF
Individual factor						
Extraversion	−0.133	0.143	−0.064	.353	.694	1.441
Neuroticism	−0.048	0.134	−0.026	.723	.626	1.597
Emotional intelligence	−0.003	0.007	−0.032	.703	.469	2.133
Optimism	0.011	0.026	0.031	.670	.878	1.139
SHS	−0.005	0.031	−0.012	.870	.655	1.526
Contextual factor						
Valence	0.527	0.185	0.173**	.005	.907	1.103
Importance	−0.003	0.033	−0.005	.938	.867	1.154
Frequency	0.153	0.108	0.083	.160	.969	1.032

*Note*. Dependent variable: inaccuracy score of level of emotional satisfaction. VIF = Variance Inflation Factor; SHS = Subjective Happiness Scale.

**p < .01.

We performed this multiple regression analysis with the inaccuracy score as dependent variable and with extraversion, neuroticism, emotional intelligence, optimism, dispositional happiness, valence (dummy coded −1 = negative; 1 = positive), importance and frequency as predictors. Table 3 contains the results of the multiple regression analyses with simultaneous predictor entries. The results revealed that the valence was the only predictor that exhibited a significant effect on the inaccuracy of affective forecasting (*p* < .01), meaning that participants differently misestimate their emotional satisfaction according to the positivity or negativity of the event. The percentage of explained variance of inaccuracy score was 4% (Model fit: *R* = .203, *R*^2^ = .041, adjusted *R*^2^ = .015).

Although the multiple regression reveals the direction of the inaccuracy score according to the predictors, the analysis does not allow us to know the details of the forecasting error. By using the inaccuracy score, the slope direction alone does not allow us to determine if the participants have under- or overestimated their affect. Indeed, one needs to take into account the predicted values of the dependent variable, as a similar slope coefficient may either translate the fact that overestimation is observed for a positive event, while underestimation holds true for negative events; or the participants could have simply underestimated to a lesser degree their affect for a positive rather than for a negative event. To better understand the direction of the impact of this last significant predictor, we examined how much intensity bias differed by experimental condition of valence.

### Analysis of Variance

**Figure 1:**
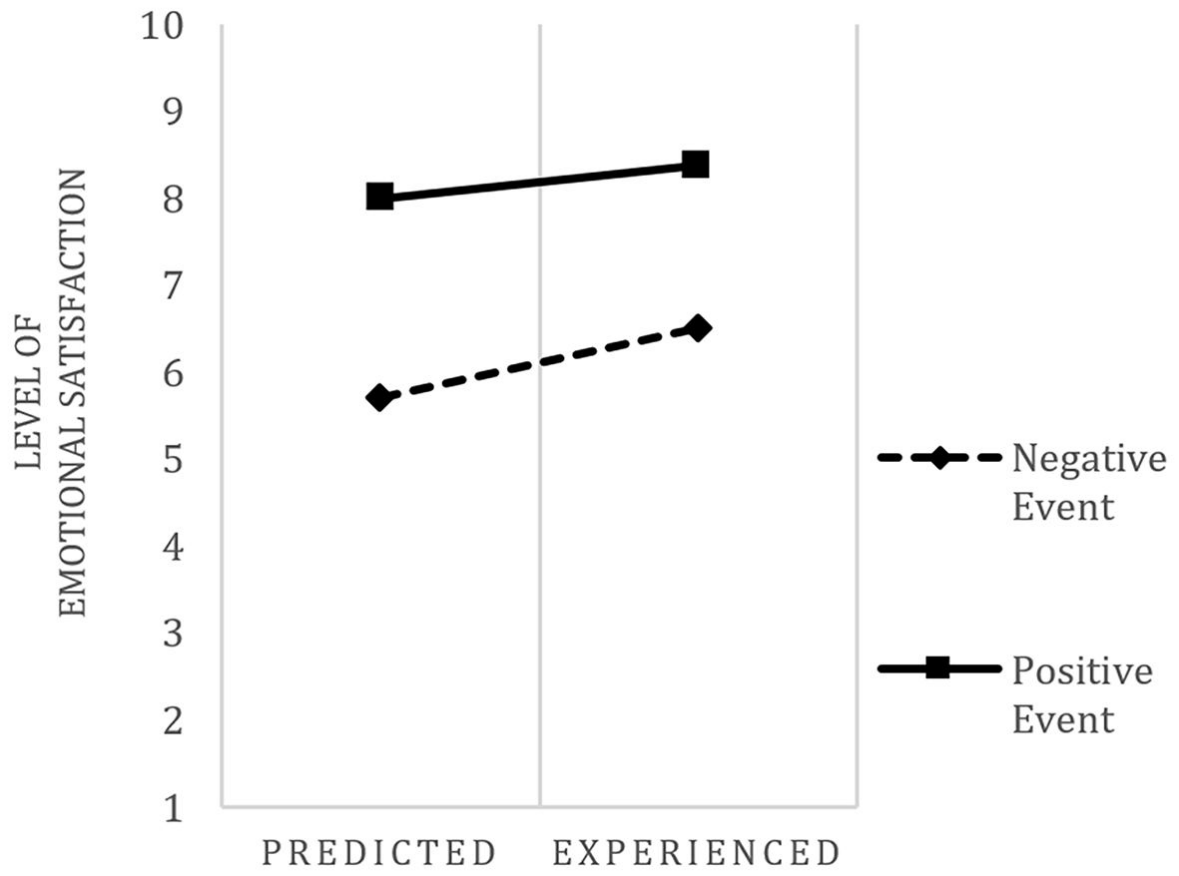
Predicted and Experienced Level of Emotional Satisfaction Rates for Events With Positive and Negative Subjective Valence

In order to evaluate the inaccuracy of participants in the absolute sense according the valence, emotional satisfaction ratings were submitted to a 2 (time: predicted vs. experienced) × 2 (valence: negative vs. positive) repeated measure ANOVA. The analysis revealed a dominant effect of time, *F*(1, 293) = 36.54, *p* < .001, and valence, *F*(1, 293) = 133.00, *p* < .001. More importantly, the analysis revealed a Time × Valence interaction showing that accuracy of affective forecasting was more sensitive to negative valence than to positive valence, *F*(1, 293) = 8.32, *p* < .01 (Figure 1). Follow up post hoc comparisons revealed that participants predicted a lower level of emotional satisfaction (*M* = 5.72, *SD* = 0.14, 95% CI [5.46, 5.99]) than what was actually experienced (*M* = 6.51, *SD* = 0.15, 95% CI [6.21, 6.82]) for negative events, *p* < .001, *d* = 0.4, 95% CI [−1.07, −0.05], and predicted lower levels of emotional satisfaction (*M* = 8.01, *SD* = 0.12, 95% CI [7.76, 8.25]) as compared to the experience (*M* = 8.29, *SD* = 0.138, 95% CI [8.02, 8.56]) for positive events, *p* < .01, *d* = 0.21, 95% CI [−0.49, −0.07]. Figure 1 reveals that in contrast to the classical pattern of intensity bias showing an underestimation of the predicted level of emotional satisfaction for negative events and an overestimation for positive events, the direction of the prediction error tends to be an underestimation of the predicted level of emotional satisfaction in both positive and negative conditions. However, the difference between predicted and actual feelings of emotional satisfaction (i.e., the inaccuracy score), was higher for the negative events (−0.79) than for the positive ones (−0.29).

## Discussion

### Affective Forecasting Accuracy

As expected, the present study showed that participants were both accurate and inaccurate to predict their feeling. According to Mathieu and Gosling (2012), the very strong association found between the participants’ prediction and experience of emotional satisfaction allows us to conclude that they were highly accurate at predicting how they will feel in *the relative sense.* This indicates that the direction of forecasts was correct: participants who predicted to feel high emotional satisfaction tended to feel high emotional satisfaction and vice-versa. However, they fail to be accurate in *the absolute sense* by misestimation of the degree to which they will feel emotionally satisfied according to event valence, congruently to previous findings in the affective forecasting literature (Gilbert, Pinel, Wilson, Blumberg, & Wheatley, 1998; Wilson et al., 2000, 2001). In addition, dispositional factors previously reported to be in relation with affective forecasting did not explain the inaccuracy of prediction: neither the participant’s level of extraversion, neuroticism, emotional intelligence, optimism or subjective happiness, nor the importance or the frequency with which the event was encountered in the past was in relation with an inaccuracy of the forecast (see Table 3). Even if multiple regression analyses enables the handling of multicollinearity between predictors, the effect of dispositional variables on the accuracy of affective forecasting could have been limited by some inter-correlations between dispositional factors (see Table 2).

The certainty of the outcome of the events could also have influenced the accuracy of the forecasts. Historically, affective forecasting was mainly studied for events with unlikely outcomes like a housing attribution (Dunn, Wilson, & Gilbert, 2003), a football match (Wilson et al., 2000) or having a date on Valentine's Day (Hoerger & Quirk, 2010). In their study about the outcome probability on affective forecasting, Buechel et al. (2014) showed through several gambling experiments that the forecasted affect is impacted by the probability of occurrence, whereas the affect experienced is more impacted by the result itself, thus inducing an inaccuracy between the affect forecasted and experienced through the degree of attention people payed to these different contextual elements. In our 100% probability condition study, the design may have allowed to reduce the general intensity bias by preventing the forecast of the affect from being influenced by a variation in probability.

### A Common Direction of Underestimation

In the present study, the valence was the only predictor of the intensity bias when predicting the level of emotional satisfaction for a personal and certain event, meaning that subjects experienced a higher level of emotional satisfaction than they had predicted 1 month earlier, both for negative and positive events (see Figure 1; for a discussion about the averages of emotional satisfaction for negative events, see SM4 in Supplementary Materials).

Even if for negative events, the intensity bias commonly predicts an underestimation of the forecasted hedonic response, the slight underestimation of the forecasted hedonic response reported in the present study for positive events is not congruent with previous findings usually showing the opposite pattern (i.e., overestimation). However, a growing part of the forecasting literature challenges the classical intensity bias direction, and more particularly through this inversion of the direction of the error for events with a positive valence (Buechel et al., 2017). For example, the demonstration of an underestimated hedonic response is increasingly observed in many contexts such as gambling games (Andrade & Van Boven, 2010; Buechel et al., 2014), fictional sad stories (Ebert & Meyvis, 2014) and social interaction or exclusion (Dunn et al., 2007; Gilbert, Lieberman, Morewedge, & Wilson, 2004; Nordgren, Bos, & Dijksterhuis, 2011). Buechel et al. (2014) suggested that the tendency for the underestimation of the hedonic intensity could be explained by the difference in richness between what is imagined and what is actually experienced. While the experience of an event outcome is vivid and replete with emotional responses, an affective prediction, in contrast, is an imagined reaction toward an abstract event. The authors explain that even if the mental simulation of an event allows to imagine how intense it will be, it is less likely to induce the same intensity of affect during the forecasting when compared to the actual experience.

In the present study, underestimations of the level of predicted emotional satisfaction for negative and positive events bring additional findings in favor of a new line of studies challenging the direction of the traditionally observed forecasting error. Together, these findings indicate that the bias is less uniform than suggested previously.

Even if the underestimation of the level of emotional satisfaction is significant for both valence conditions (negative: *p* < .001, *d* = 0.4, 95% CI[−1.07, −0.05], positive: *p* < .01, *d* = 0.21, 95% CI[−0.49, −0.07]), it can be noticed that the size effect is stronger for negative than for positive events (i.e., moderate effect for negative and low effect for positive). In addition, the positive relationship found between the forecasting inaccuracy and the valence in the multiple regression analyses can be explained by a stronger underestimation of emotional satisfaction for negative than for positive events, which supports previous findings highlighting the prevalence of the negative valence on the intensity bias for life events (The stronger value of the effect observed for negative events is discussed through memory bias and motivational theories in SM5, in Supplementary Materials) (e.g., Feys & Anseel, 2015; Finkenauer et al., 2007; Loewenstein, 2007).

Before drawing any conclusions, it is necessary to put forward several limitations of the study in order to more critically interpret its results. The main strength of this study is at the same time its greatest weakness: the diversity of events used for forecasting by the participants. Although this methodological choice allowed to provide a higher variability within responses among the many studied factors, and guaranteed that subjective valence indeed went in the intended direction, it nonetheless limited the scope of our conclusions on the resulting relationships between variables. As a result, interpretation of results must be carried out with utmost caution. In the future, the use of personal life events limited to fixed predefined categories (e.g., health, family or professional events) could be a methodological compromise to more solidly back up the present data. Secondly, the procedure was thought up to limit the impact of occurrence probability of the event through the instruction to forecast only for an event for which the outcome was absolutely certain. An interesting question for future research would be to use a similar multifactorial procedure by including groups of events with different levels of probability of occurrence. Finally, following the robustness of the negative effect found in this study and throughout the literature, we suggest that future studies should include the valence as a continuous variable rather than a categorical one.

## Conclusion

These findings suggest that among the main dispositional and contextual variables previously studied in the literature, the subjective valence of the event is the stronger predictor of inaccuracies in affective forecasting. In addition, our findings showed a stronger underestimation of the forecast for negative life events compared to positive ones which can be considered as low, representing a negative valence effect. We found an underestimation of the emotional satisfaction both for the positive and negative events, that is, a classical direction of misestimation for negative events but a reversal of direction for the misestimation of positive ones. Congruently to a growing body of literature in affective forecasting, our results challenge the direction of the traditional intensity bias. Finally, the results of the present study support that people can be both accurate in the direction of the forecast of their affect and inaccurate in the absolute intensity of their prediction according the valence of the predicted event.

## Notes

For this article the following Supplementary Materials are available via the PsychArchives repository (for access see Index of Supplementary Materials below):

- SM1: Additional design information.
- SM2: Detail about the choice of the affective target.
- SM3: Metric issue in affective forecasting literature.
- SM4: Discussion about the averages of emotional satisfaction for negative events.
- SM5: Discussion about the stronger value of the effect observed for negative events through memory bias and motivational theories.
